# T cell plasticity in renal autoimmune disease

**DOI:** 10.1007/s00441-021-03466-z

**Published:** 2021-05-03

**Authors:** Shiwa Soukou, Samuel Huber, Christian F. Krebs

**Affiliations:** 1 Hamburg Center for Translational Immunology (HCTI), University Medical Center Hamburg-Eppendorf, Hamburg, Germany; 2 I. Department of Medicine, University Medical Center Hamburg-Eppendorf, Hamburg, Germany; 3 Translational Immunology, III. Department of Medicine, University Medical Center Hamburg-Eppendorf, Hamburg, Germany

**Keywords:** Renal autoimmune disease, Th17 cells, T cell

## Abstract

The presence of immune cells is a morphological hallmark of rapidly progressive glomerulonephritis, a disease group that includes anti-glomerular basement membrane glomerulonephritis, lupus nephritis, and anti-neutrophil cytoplasmic antibody (ANCA)–associated glomerulonephritis. The cellular infiltrates include cells from both the innate and the adaptive immune responses. The latter includes CD4^+^ and CD8^+^ T cells. In the past, CD4^+^ T cell subsets were viewed as terminally differentiated lineages with limited flexibility. However, it is now clear that Th17 cells can in fact have a high degree of plasticity and convert, for example, into pro-inflammatory Th1 cells or anti-inflammatory Tr1 cells. Interestingly, Th17 cells in experimental GN display limited spontaneous plasticity. Here we review the literature of CD4^+^ T cell plasticity focusing on immune-mediated kidney disease. We point out the key findings of the past decade, in particular that targeting pathogenic Th17 cells by anti-CD3 injection can be a tool to modulate the CD4^+^ T cell response. This anti-CD3 treatment can trigger a regulatory phenotype in Th17 cells and transdifferentiation of Th17 cells into immunosuppressive IL-10-expressing Tr1 cells (Tr1exTh17 cells). Thus, targeting Th17 cell plasticity could be envisaged as a new therapeutic approach in patients with glomerulonephritis.

## Introduction

Glomerulonephritis (GN) is a heterogeneous group of diseases that cause immune-mediated injury of the renal glomerulus. The most aggressive form of glomerulonephritis, which can result in rapid decline in kidney function and shows glomerular necrosis, crescent formation, and immune cell invasion, including glomerular and periglomerular T cell infiltration is referred to as crescentic glomerulonephritis (cGN). The cause of cGN is a heterogeneous group of immune-mediated diseases that is classified into three types according to Couser (1988). Type I is driven by antibodies against the basal membrane as observed in anti-glomerular basement membrane glomerulonephritis (anti-GBM GN) or the Goodpasture syndrome (anti-GBM with pulmonary involvement). Type II is mediated by the accumulation of immune complexes, which is regularly seen in lupus nephritis (Tipping and Holdsworth 2006). Type III, the most common form of cGN, is histologically characterized by immune cell infiltration but absence of antibody depositions. Therefore, these entities have been termed pauci-immune glomerulonephritis. This form of cGN is associated with anti-neutrophil cytoplasmic autoantibodies (ANCA) (Couser 2012). All of these types of cGN cause intra-capillary and extra-capillary proliferation and infiltration with various types of immune cells (Janssen et al. 1998; Kurts et al. 2013).

The immune system is composed of different immune cells such as B cells, monocytes, and natural killer (NK) cells and of CD8^+^ and CD4^+^ T cells (Brodin and Davis 2017). Under physiological conditions, pro- and anti-inflammatory processes are balanced in order to maintain homeostasis (Foussat et al. 2003; Harrison et al. 2019). However, in autoimmune diseases, perturbations and imbalances in the immune cell composition and function can cause destructive inflammatory processes, which can result in glomerulonephritis (Lin and Zhang 2017) in the kidney. The concept of T cell involvement in the immunopathogenesis of glomerulonephritis is supported by MHC association studies in patients with ANCA-associated GN (Bonatti et al. 2014). In particular, CD4^+^ T cell responses are critically involved in immune-mediated tissue injury.

CD4^+^ T cells can be subdivided into different pro-inflammatory and anti-inflammatory subsets. Th1, Th2, and Th17 cells provide protection against exogenous offending agents but are also involved in cell-mediated autoimmunity. Regulatory T cells, such as Foxp3^+^ regulatory T cells (Treg cells) and type 1 regulatory T cells (Tr1 cells), prevent autoimmune reactions and overwhelming effector responses. Importantly, the concept of stable lineage commitment of these T cell subsets has been challenged by publications that indicate alterations of the cytokine and transcriptional profile in T cells (Cano-Gamez et al. 2020; Gagliani et al. 2015; Hirota et al. 2011; Kiner et al. 2021). Th17 cells have been reported to be the main driver of immune-mediated diseases, and this T cell subset can have a high degree of plasticity, in that it can convert into other pro-inflammatory and anti-inflammatory subsets. However, the mechanisms that drive plasticity versus stability and their relevance in autoimmunity are incompletely understood. Further insight will be key to develop novel therapeutic strategies targeting Th17 cell plasticity in autoimmunity (Krebs and Panzer 2018).

Autoimmune diseases can affect several organs, such as the central nervous system (CNS) in multiple sclerosis (MS), the intestine in inflammatory bowel disease (IBD), the joints in rheumatoid arthritis, and the kidneys in glomerulonephritis. However, what triggers the development of such autoimmune diseases is not yet completely understood. Genetic predisposition and spontaneous mutations have been described as influencing disease initiation and progression in affected patients. In addition, microbial and environmental factors have been reported to contribute to the pathological conditions (Begue et al. 2011; Engelhardt and Grimbacher 2014; Glocker et al. 2010; Xue and Mei 2019; Zhu et al. 2017).

The critical impact of Th1 and Th17 cells in the pathogenesis of glomerulonephritis has been demonstrated by our group and others (Krebs et al. 2013; Paust et al. 2009; Pisitkun et al. 2012; Summers et al. 2009). Interestingly, the shift from a Th17 dominated immune response at early stages of GN to a dominance of Th1 cells at a later time point raised the question of potential plasticity of Th17 cells and, specifically, whether they have the ability to convert into Th1 phenotypes (Paust et al. 2012).

The knowledge of T cell plasticity and stability mainly derives from animal models. However, the emergence of single-cell transcriptomics allows transcriptional states to be addressed in a comprehensive and unbiased fashion. From these investigations, developmental trajectories could be inferred that suggest transdifferentiation of T cells. Single-cell omics is covered in another article by Zhao et al. in this issue of the journal.

In this article, we will review the current literature on Th17 cell plasticity with a focus on immune-mediated kidney diseases. Moreover, we will discuss the role of pathogen-induced Th17 cells and potential therapeutic options.

### CD4^+^ T cell subsets

T cells play an important role in host defense and clearance of pathogens. T cells develop in the thymus and express unique T cell receptors (TCRs). While the majority of T cells express TCRs with ⍺ and β chains (conventional T cells), others carry TCRs with a ɣ chain and a δ chain (ɣδ T cells).

Conventional T cells are separated into two groups which are defined by either CD8 or CD4 expression. CD8^+^ T cells, also referred to as cytotoxic T cells, respond to antigens presented by the MHC-I group, which are expressed on nucleated cells. These cells are mainly involved in cancer and virus elimination.

In contrast, CD4^+^ T cells are activated when they recognize antigens in the TCR, MHC-II, and peptide complex in their interaction with antigen-presenting cells (APCs) such as dendritic cells. This activation gives rise to cell differentiation into various CD4^+^ T cell subsets that orchestrate the immune response by secreting cytokines and other factors. These various CD4^+^ T cell subsets can exhibit both pro-inflammatory and anti-inflammatory functions.

The first description of different subsets of CD4^+^ T helper cells was published by Mosmann and Coffmann in 1986 (Mosmann et al. 1986). They have identified two types of T helper cells (Th cells) that are distinguished by their cytokine and transcription factor profiles. These cells were referred to as Th1 and Th2. Th1 cells are characterized by the expression of the transcription factor T-bet and the production of the cytokines such as IL-12, the tumor necrosis factor-alpha (TNF-⍺), and IFN-ɣ (O'Shea and Paul 2010; Szabo et al. 2000). During Th1 cell differentiation, binding of IL-12 to its receptor plays a fundamental role (Kitching et al. 2005) by activating the signal transducer and activator of transcription-4 (STAT4). This can also result in the expression of IL-10 by Th1 cells (Neumann et al. 2014). T-bet, the master transcription factor of Th1 cells, is required for expression of IFN-ɣ in most of the T cell subsets (Thieu et al. 2008; Yang et al. 2020). Together, IFN-ɣ and T-bet drive the activation of STAT1 which is important for the maintenance of the Th1 phenotype by upregulating the IL-12Rβ2 subunit (Afkarian et al. 2002; Kitching et al. 2005). The secretion of IFN-ɣ can even be enhanced by IL-12 expression together with IL-18 (Nakanishi et al. 2001). IFN-ɣ-expressing T cells serve important functions in the host defense, in particular in the immune response against intracellular pathogens such as *Mycobacterium tuberculosis* or *Mycobacterium lepromatosis* (Martinez-Barricarte et al. 2018; Yang et al. 2020). Th1 cells activate phagocytes, allowing infected cells to be eliminated and the anti-microbial response to be supported (Romagnani 1999). In addition, Th1 cells also have a protective capacity against viral infection by their migration to sites of inflammation and cytokine expression (Maloy et al. 2000).

The signature cytokines produced by Th2 cells are IL-4, IL-5, IL-9, and IL-13. Furthermore, Th2 cells are able to secrete IL-10 (Mosmann and Moore 1991). By upregulating IL-10, Th2 cells can inhibit Th1 cells by dampening IFN-ɣ secretion (Mosmann and Moore 1991). IL-4 along with IL-2 is necessary for the differentiation of Th2 cells (Le Gros et al. 1990). To this end, the binding of IL-4 to its receptor results in an activation of the STAT6, which is important for the expression of the subset-specific transacting T cell–specific transcription factor GATA3 (Kaplan et al. 1996; Zheng and Flavell 1997).

Generally, Th2 cells play a fundamental role during infections with extracellular parasites like *Nippostrongylus brasiliensis* (Ozawa et al. 2005) or *Schistosoma mansoni* (Mosmann and Moore 1991). The release of IL-5 and IL-13 by Th2 cells can induce eosinophils which result in protection against parasites by pushing infected cells into apoptotic states (Martinez-Moczygemba and Huston 2003). In addition to these protective effects, Th2 cells are also involved in airway inflammation (Woodruff et al. 2009). Accordingly, many subtypes of asthma are associated with the abundance of Th2 cells in the lung.

Furthermore, other CD4^+^ T cell subsets have been identified in the past decade such as IL-9-expressing Th9 cells, IL-22-expressing Th22 cells, and follicular T helper cells (Tfh cells). However, the most prominent of those additional subsets might be Th17 cells, which are effector cells distinct from Th1 and Th2 cells (Harrington et al. 2005). Th17 cells express the transcription factor, ROR-ɣt, and secrete high levels of their signature cytokines IL-17A and IL-17F (Ivanov et al. 2006; Krummey et al. 2014). Usually, Th17 cells fight against pathogens; however, Th17 cells have been reported to drive autoimmune inflammation in the CNS, the skin, the intestine, and the kidneys (Esplugues et al. 2011; Krebs et al. 2016a; Langrish et al. 2005; Lowes et al. 2008; Park et al. 2005). In many conditions, Th17 cell proliferation and effector cytokine production can be controlled by Foxp3^+^ regulatory T cells and type 1 regulatory T cells (Tr1), which do not express Foxp3 (Diefenhardt et al. 2018; Huber et al. 2011). These cells function as regulatory cells by suppressing effector cell proliferation and thereby restoring immune homeostasis. An important cytokine in this context is IL-10 that is mainly produced by regulatory T cells. The main focus of the next sections will be on the literature surrounding the T cell subsets, Th17 cells, and regulatory T cells since they are of great importance during glomerulonephritis and are very promising as potential therapeutic targets.

### Th17 cell development and biology

Th17 cells can be induced both in vitro and in vivo by stimulating TCR in the presence of specific cytokines (Ivanov et al. 2006). In mice and humans, IL-6 and transforming growth factor beta (TGF-β) are described as the drivers in Th17 cell development (Bettelli et al. 2006; Manel et al. 2008; Veldhoen et al. 2006). Although IL-23 does not seem to be a main driver of Th17 cell differentiation, it is reported to play an important role in their proliferation and maintenance (Bettelli et al. 2006; Veldhoen et al. 2006). Th17 cells are known to be induced by IL-6, IL-1β, and IL-23 (Langrish et al. 2005; Lee et al. 2020), and this cytokine combination gives rise to more pathogenic Th17 cells. Some Th17 cells polarized in the presence of IL-1β and IL-23 produce high levels of IL-22 (Chung et al. 2009). Recently, it was reported that IL-22-expressing Th17 cells produce high levels of IFN-ɣ. These Th17 cells display a Th1-like phenotype and fulfill characteristics of pathogenic Th17 cells that strongly contribute to inflammation (Omenetti et al. 2019).

In contrast to these pathogenic Th17 cells, the combination of IL-6 and TGF-β is reported to induce, in part, non-pathogenic Th17 cells which can produce IL-10 (McGeachy et al. 2007). This IL-10 secretion under Th17 polarizing conditions is regulated by c-musculoaponeurotic fibrosarcoma (c-Maf), which in turn is induced by IL-6 and TGF-β (Xu et al. 2009). Interestingly, RORɣt, the master transcription factor of Th17 cells, also represses IL-10 production in Th17 cells and thereby sustains their effector function in intestinal inflammation (Sun et al. 2019).

In addition to cytokine production, differences between Th17 subsets can be observed at the transcriptional level (Lee et al. 2020). The gene signature of non-pathogenic or physiological Th17 cells is described as highly enriched for genes such as *Maf*, *Ahr*, and *IL-10* (Lee et al. 2020). Pathogenic Th17 cells, on the other hand, express high levels of *Csf2*, *Tbx21*, and *Gzmb* (Lee et al. 2020).

IL-17A is described as a cytokine that is important for the fortification of the epithelial barrier in order to protect the host from pathogen invasion (Lee et al. 2015). Thereby, it has a crucial role in activating the innate immune system by recruiting neutrophils, for example (Disteldorf et al. 2015; Korn et al. 2009). Furthermore, Th17 cells that co-express IL-17A and IL-22 cells produce β-defensin, which is important for the secretion of anti-microbial peptides by epithelial cells (Liang et al. 2006). These anti-microbial peptides are important for host defense against bacterial infections. It is important to note that the Th17 immune response plays a critical role in maintaining the gut homeostasis. In this context, the reaction to antigens from invasive intestinal bacteria such as segmented filamentous bacteria (SFB), *Escherichia coli* O157, and *Citrobacter rodentium* induces intestinal Th17 cells (Atarashi et al. 2015; Ivanov et al. 2009; Sano et al. 2015). The bacterium *Citrobacter rodentium* is a well-described intestinal pathogen that causes tissue damage driven by proliferation of Th17 cells with inflammatory potential (Omenetti et al. 2019). Th17 cells display a protective role against *Citrobacter rodentium* infections. Although it is not the main cytokine that drives Th17 cell differentiation, IL-23 strongly drives Th17 function to fight against this pathogen (Mangan et al. 2006). Therefore, the natural presence of Th17 cells in the small intestine allows a fast Th17 cell response after infection. In addition to bacteria, fungi such as *Candida albicans* can induce Th17 cells (Acosta-Rodriguez et al. 2007; Sallusto 2016; Zielinski et al. 2012) and Th17 cell immune response and related cytokines have recently also been implicated in the immune response to SARS-CoV-2 (De Biasi et al. 2020; Zhao et al. 2021).

Although physiological Th17 cells are important for the maintenance of gut homeostasis and barrier integrity, the immune system can also give rise to pathogenic Th17 cells that contribute to pathological inflammation in intestinal and extra-intestinal diseases, including multiple sclerosis (Langrish et al. 2005), colitis (Lee et al. 2009), and glomerulonephritis (Kitching et al. 1999; Steinmetz et al. 2011).

**Fig. 1:**
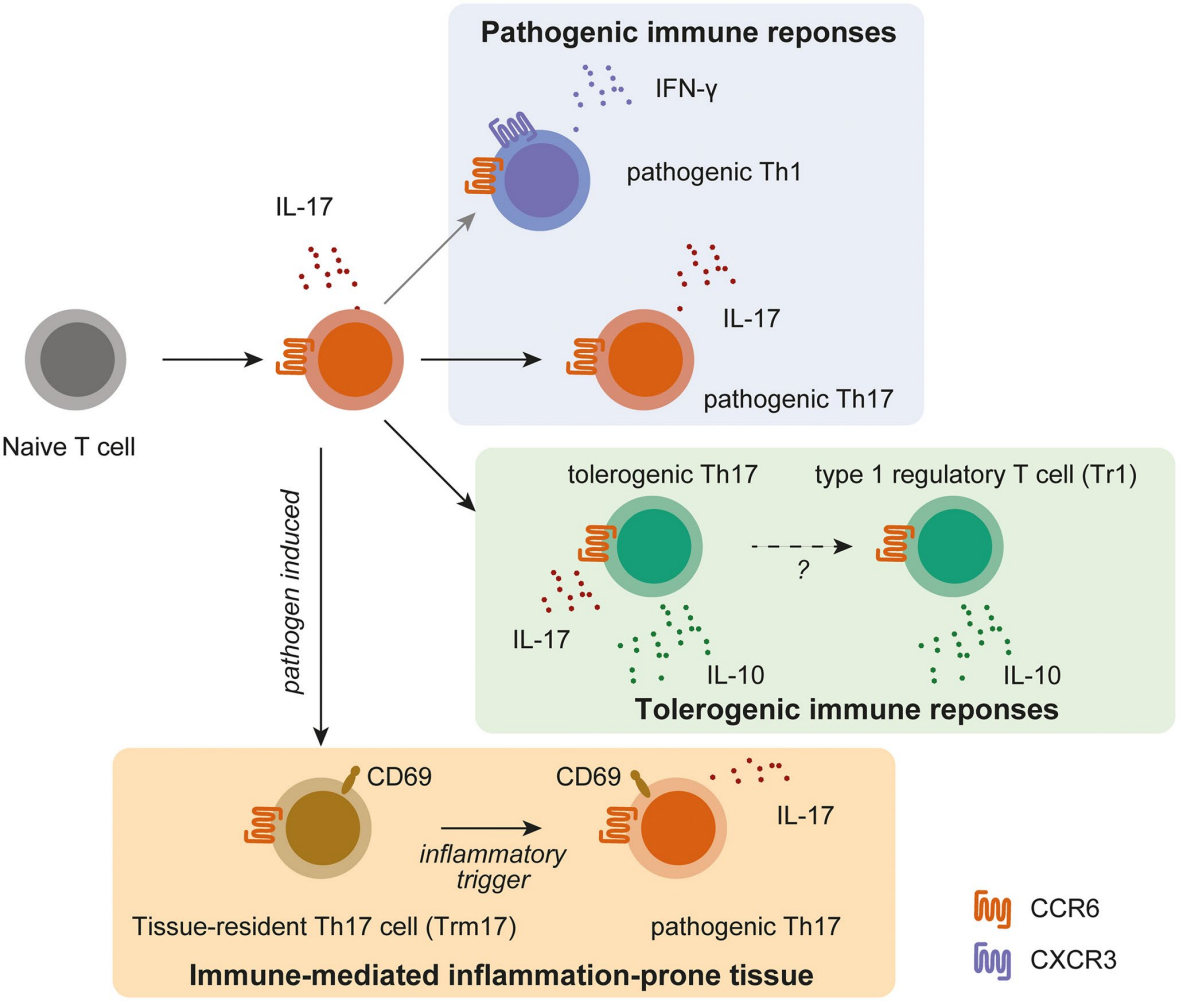
Overview of potential differentiation routes of renal Th17 cells. IL-17-producing Th17 cells (red) can present a low degree of plasticity in experimental glomerulonephritis, and transdifferentiation into IFN-gamma-producing Th1 cells (blue) is less common in the kidney compared to findings in the inflamed central nervous system or in the intestine. Anti-CD3 treatment can induce IL-10-producing Th17 cells with a tolerogenic phenotype (green). Pathogen-induced Th17 cells develop into tissue-resident cells (Trm17) that can be activated by unspecific triggers and contribute to tissue damage in immune-mediated glomerulonephritis

Interestingly, renal Th17 cells in glomerulonephritis are related to Th17 cells in the intestine, as suggested by data coming from experimental glomerulonephritis (Krebs et al. 2016a) (Fig. 1). C–C motif chemokine receptor (CCR)-6 guides Th17 cells into the small intestine and the kidney (Esplugues et al. 2011; Krebs et al. 2016a). Interestingly, Th17 cells have the capacity to emigrate from the intestine in a S1PR1-dependent fashion and to migrate into the inflamed kidney, where they contribute to renal tissue damage in experimental GN (Krebs et al. 2016a).

### Th17 cell plasticity

Originally, every CD4^+^ T cell subset was assumed to be stable after differentiation (Mosmann et al. 1986). This view of the T cell immune response has, however, been challenged in the last decade, and it is now accepted that T cells can display a high degree of plasticity. These T cells can actually change the expression of transcription factors and cytokines within one lifecycle (Gagliani et al. 2015; Wang et al. 2014). This change can be transient when cells co-express cytokines. However, T cells can lose their transcriptional and cytokine signature and fully switch to another T cells subset, a process named transdifferentiation (Akamatsu et al. 2019). This phenotypical change can occur spontaneously, but it can also be influenced in vitro and in vivo. Therefore, T cell priming and the changes in the micro-environment can modulate T cell function and result in T cell plasticity (Nistala et al. 2010; Omenetti et al. 2019). The molecular network that drives T cell plasticity is still incompletely understood. However, a progressive adaptation to the tissue may play an important role (Miragaia et al. 2019). The concept of transdifferentiation is challenged by some authors as they postulate a phenotypic continuum of T cell subtypes (Kiner et al. 2021).

In the case of Th17 cells, transdifferentiation has been described as multidimensional in terms of cell fate. Th17 cell transdifferentiation can result in diverse functions. During EAE, some IFN-ɣ-producing cells are described as originating from Th17 cells (named exTh17 cells) (Gagliani et al. 2015; Hirota et al. 2011). T cell transdifferentiation has been observed to promote the pathogenic properties of the cells within the host (Hirota et al. 2011; Komatsu et al. 2014). Likewise, *Citrobacter*-induced Th17 cells are highly plastic and mainly drive inflammation by expressing additional IFN-ɣ (Omenetti et al. 2019). In contrast, Th17 cells in the small intestine tend to upregulate IL-10 during transient gut inflammation induced by TCR-stimulation (Gagliani et al. 2015). Importantly, anti-inflammatory IL-10-producing Th17 cells can be converted in pro-inflammatory cells under the influence of IL-1ß (Noster et al. 2016; Zielinski et al. 2012). IL-10 upregulation is described as being driven by the transcription factor c-Maf that supports IL-27 driven IL-10 production in Th17 cells (Chang et al. 2017). A second antigen stimulation of human Th17 cells gives rise to anti-inflammatory Th17 cells expressing high levels of c-Maf, while pro-inflammatory Th17 cells do not upregulate c-Maf in this context (Aschenbrenner et al. 2018).

In addition to IL-10 and IL-17A co-expression, some Th17 cells have the ability to transdifferentiate into Tr1 cells. Those cells are termed Tr1^exTh17^ cells. Here, TGF-β and Ahr are described as the main drivers of this conversion (Gagliani et al. 2015). Tr1^exTh17^ cells mainly display a strong regulatory phenotype. Many factors favoring T cell plasticity have been described. Both T cell priming and the environment can drive T cell conversion (Omenetti et al. 2019). T cell conversion from effector cells expressing IL-10 has been described in the context of self-limitation (Neumann et al. 2014). Converted cells keep some characteristics of the effector cells from which they originated, such as mild expression of the CCR6 that guides pro-inflammatory and anti-inflammatory T cells to sites of inflammation. Furthermore, low levels of RORɣt can be detected in exTh17 cells converted into Tr1 cells (Gagliani et al. 2015). Although these cells express chemokine receptors and transcription factors to a lesser extent than Th17 cells, the origin of the cell still potentially influences cell functions. During functional in vivo experiments, in comparison to conventional Tr1 cells that originate from naïve CD4^+^ T cells, both cells exhibit the same capacity to regulate colitis development (Gagliani et al. 2015).

The phenotype of effector Th17 cells with regard to their potential plasticity is dependent on the local micro-environment, defined by different tissues and inflammatory triggers (Krebs et al. 2016b). We are only beginning to understand what factors make up the micro-environmental factors that go beyond cytokines and cell–cell interactions, potentially including glucose levels, oxidative stress, fatty acids, and many more. Several publications from the past years have provided strong evidence that the local salt concentration critically impacts Th17 cells (Kleinewietfeld et al. 2013; Muller et al. 2019; Wu et al. 2013). Importantly, high salt concentrations can push either pro-inflammatory or anti-inflammatory Th17 cell (Luo et al. 2016; McGeachy et al. 2007) and the local cytokine milieu can control these two contradictory cell types (Matthias et al. 2020). Interestingly, renal Th17 cells in mice with experimental GN display a more stable phenotype as compared to Th17 cells in the inflamed CNS in experimental autoimmune encephalomyelitis (EAE) (Krebs et al. 2016b). When FACS-sorted, Th17 cells are investigated in experimental glomerulonephritis in transfer models, or IL-17A^CRE^ × Rosa26^YFP^ fate reporter mice are used to track Th17 cells over time, about 30% of these Th17 cells shut down their capacity to express IL-17A (Krebs et al. 2016b). Interestingly, additional TCR-stimulation treatment with CD3-specific antibodies resulted in production of IL-10 in Th17 cells, and these treated mice showed less glomerular and tubulointerstitial kidney damage (Krebs et al. 2016b).

As stated before, Th17 cells can be induced by different infectious triggers. However, until recently, it was known whether the infection-induced Th17 cells can also promote immune-mediated tissue injury. Interestingly, we identified that bloodstream infections by *Staphylococcus aureus* and *Candida albicans* and urinary tract infections by *Escherichia coli* can induce prominent Th17 immune responses, particularly in the kidney (Krebs et al. 2020). After these infections are cleared, Th17 cells become tissue resident and remain in the kidney over a long period of time. Interestingly, these tissue-resident Th17 cells (termed Trm17) can aggravate tissue injury in models of experimental glomerulonephritis. Of note, in terms of antigens, the antigens in experimental GN and the infectious triggers inducing these cells are very distinct. Single-cell RNA-sequencing revealed the major subsets that maintain the potential to express IL-17A and F of these Trm17 cells and revealed their profiled expression.

Based on these findings, different T cells subsets can potentially switch their phenotype from effector functions in infection to tissue-resident functions. These T cells can acquire innate-like functions and react to stimuli by cytokine expression in a TCR-independent manner. These T cells might be protective in infections but become pathogenic if immune-mediated tissue inflammation is established. Finally, these tissue-resident memory T cells in the kidney are a potential therapeutic target in patients with chronic remitting inflammation as seen in ANCA-associated vasculitis.

Collectively, these findings show that T cell differentiation is not a dead end in cellular development, but rather a temporary condition that can be influenced and pushed into regulatory programs by triggering the corresponding pathways.

### Generation and features of regulatory T cells

Regulatory T cells are necessary to control the immune response. There are two major subsets which show a strong capacity to suppress effector T cells: FoxP3^+^ regulatory T cells (T reg cells) and type 1 regulatory T cells (Tr1 cells). In both types of regulatory T cells, IL-10 plays a major role in cell maintenance and, thereby, in sustaining homeostasis (Brockmann et al. 2017). Foxp3^+^ Treg cells and Tr1 cells have the potential to suppress Th17 and Th1 cells directly via IL-10, which has been shown in the intestine (Huber et al. 2011).

Within the group of regulatory T cells, Treg cells are defined by the expression of its master transcription factor Foxp3 (Fontenot et al. 2005) and, indeed, Foxp3 expression is essential for the differentiation and maintenance of these immunosuppressive cells. On their surface, Foxp3^+^ Treg cells express the IL-2 receptor ⍺ chain (Chinen et al. 2016). The activation of Foxp3 depends on IL-2 and TGF-β signaling (Chen et al. 2003; Zorn et al. 2006). These two cytokines are described in mice and humans and in the in vitro generation of Foxp3^+^ Treg cells. The important role of Foxp3^+^ Treg cells is shown when patients lack the gene, Foxp3, which results in the development of a fatal autoimmune disease (Bennett et al. 2001).

During inflammatory conditions, functional Foxp3^+^ Treg cells exhibit a strong suppressive potential to inhibit Th17 and Th1 cells during colitis and glomerulonephritis, respectively (Huber et al. 2011; Kluger et al. 2014; Ostmann et al. 2013). Furthermore, in mice and humans, Foxp3^+^ Treg cells were shown to significantly reduce kidney damage and to support acceptance of a transplant kidney by the host (Berglund et al. 2013; Savage et al. 2018). Endogenous IL-10 and IL-10 derived from Foxp3^+^ Treg cells is reported to ameliorate crescent formation, by modulating Th1 and Th17 cell responses (Kitching et al. 2000; Ostmann et al. 2013).

Tr1 cells are induced in the periphery and have been identified as potent suppressors of the immune system. They were originally defined based on the lack of Foxp3 expression and high expression of IL-10 (Roncarolo et al. 2014). Furthermore, Tr1 cells produce moderate levels of TGF-β and IFN-ɣ. However, the cytokines IL-4, IL-2, and IL-17A/F can also be expressed at very low levels by Tr1 cells (Gagliani et al. 2013). Originally, IL-10 was assumed to induce Tr1 cell generation. Until now, the role of IL-10 was shown during the regulation of stability and continuous IL-10 production by Tr1 cells (Brockmann et al. 2017). However, IL-27 has been identified to strongly induce the generation of Tr1 cells by the interaction of aryl hydrocarbon receptor (AHR) and c-Maf (Apetoh et al. 2010). Furthermore, in vitro, IL-27 and TGF-β induce high numbers of Tr1 cells (Apetoh et al. 2010).

In contrast to other T cell subsets, there has not yet been a single transcription factor shown to be responsible for Tr1 cell differentiation. Nonetheless, various transcription factors are under discussion as to their involvement in Tr1 cell biology. These include Eomesodermin (Eomes) (Zhang et al. 2017), liver X receptor (LXR) (Brockmann et al. 2018), and early growth response 2 (Egr-2) (Okamura et al. 2009) in addition to c-Maf and PR domain zinc finger protein 1 (Blimp-1) (Chihara et al. 2018).

As described previously, Tr1 cells can originate from naïve CD4^+^ T cells. Interestingly, recent investigations show that they can also originate from former effector T cells (Gagliani et al. 2015). This generation was observed mainly in the small intestine when mice were injected with anti-CD3 antibody, which induces high numbers of suppressive Tr1 cells (Kamanaka et al. 2006). In this context, the generation of Tr1 cells from Th17 cells is dependent on TGF-β and on the AHR (Gagliani et al. 2015).

The administration of the anti-CD3 antibody leads to a strong induction of IL-17A in all parts of the small intestine (Esplugues et al. 2011). Furthermore, high numbers of regulatory cells are induced that have the ability to inhibit Th17 proliferation (Huber et al. 2011; Perruche et al. 2008). Tr1 cells play an important role in maintaining homeostasis during gut inflammation. First described in 1997, they have already been defined to be antigen-specific and successful in preventing colitis (Groux et al. 1997). The specific potency of Tr1 cells depends on their ability to produce and respond to IL-10 (Brockmann et al. 2017; Huber et al. 2011; Yao et al. 2015). IL-10 is a cytokine mostly associated with suppressive function. Regulation of pro-inflammatory cell types via IL-10 has been shown to be beneficial in healthy patients under homeostatic conditions. Also, in patients suffering from colitis or cGN, treatment with IL-10 or Tr1 cell–enriched CD4^+^ T cell cocktail has strong potential to inhibit inflammatory responses (Desreumaux et al. 2012; Mfarrej et al. 2017; Petrelli et al. 2015; Soranno et al. 2016). Generally, in mice and humans that lack Tr1 cells, the development of spontaneous colitis can be observed (Engelhardt and Grimbacher 2014; Scheinin et al. 2003). Furthermore, when colitogenic mice are treated with IL-10, decreased levels of Th17 cells and IFN-ɣ^+^ Th17 cells can be observed (Huber et al. 2011).

Previously, it was shown that anti-CD3 antibody treatment ameliorates crescent formation and tubular damage during glomerulonephritis when administrated early after disease induction (Krebs et al. 2016b). Furthermore, anti-CD3 antibody treatment increases IL-10 production in all analyzed T cell subsets. CD3-specific antibody treatment induces a rapid activation induced cell death which is also accompanied by a cytokine storm (Penaranda et al. 2011). Foxp3^+^ regulatory T cells and Th17 cells are also activated. However, these cells are more resistant to activation induced cell death (Penaranda et al. 2011; Shi et al. 2009; Yu et al. 2017). Furthermore, TGF-β1 and IL-6, which are produced by phagocytes upon phagocytosis of the dead cells, further promote the expansion of the Foxp3^+^ Treg and Th17 cells (Bettelli et al. 2006; Chung et al. 2006; Fadok et al. 1998; Veldhoen et al. 2006; Zheng et al. 2007). Finally, Th17 cells, which are CCR6-positive, are recruited via IL-17A induced CCL20 production by intestinal epithelial cells to the small intestine. Interestingly, Foxp3^+^ and Foxp3^−^ IL-10-producing T cells are likewise recruited to the small intestine and thereby limit intestinal inflammation and tissue damage (Huber et al. 2011; Kamanaka et al. 2006). Accordingly, in the small intestine, the treatment induces only transient inflammation (Clayburgh et al. 2005). In line with these data, increased frequencies of IL-10 producing CD4^+^ T cells were observed. Of note, a protective effect of anti-CD3 antibody treatment was observed when mice were treated on days 6 and 8 after glomerulonephritis induction (Krebs et al. 2016b).

In line with the abovementioned data, previous publications have described SFB-induced Th17 cells as tissue resident and described them as not contributing to inflammation (Omenetti et al. 2019). In the gut, the Th17 cells actually exhibited a high level of plasticity; as a result of this, IFN-ɣ-producing Th17 cells were present and these showed enhanced metabolic activity (Omenetti et al. 2019). Other studies have also described Th17 cells as showing high metabolic activity and as capable of converting to Th1 (Karmaus et al. 2019). It might be possible that in glomerulonephritis, a fraction of exTh17 cells actually convert to Th1 cells. Those cells might have additionally outranked the possible protective effect of the anti-CD3 antibody, and thus, protection could not be detected.

These findings indicate that similar T cells can display a different behavior and plasticity according to the micro-environment provided by different tissues.

## Concluding remarks

Over the past decade, the way we view different T cell subsets has shifted substantially, largely on account of genetic models that allow the sorting of pure populations and the tracking of cell fates over time. Examples of these models are acute reporter mice and fate reporter mice. While initial observations suggested a terminally differentiated cell state within each subset, there can be substantial plasticity in the T cell compartment. Importantly, T cells have the ability to turn up and down effector functions and to differentiate into distinct effector types, but they can also acquire an immunosuppressive function.

The better understanding of the molecular mechanisms of T cell plasticity that has evolved over the past decade opens new avenues in treating immune-mediated diseases. By modulating T cell immune responses in a disease specific and a tissue-specific manner, detrimental T cell effector function could be converted into regulatory effects and therefore reduces immune-mediated inflammation. However, further studies are needed before these therapeutic concepts can be translated into clinical trials in the field of autoimmune kidney diseases.
